# A Health Technology Assessment in Maxillofacial Cancer Surgery by Using the Six Sigma Methodology

**DOI:** 10.3390/ijerph18189846

**Published:** 2021-09-18

**Authors:** Carlo Ricciardi, Giovanni Dell’Aversana Orabona, Ilaria Picone, Imma Latessa, Antonella Fiorillo, Alfonso Sorrentino, Maria Triassi, Giovanni Improta

**Affiliations:** 1 Department of Electrical Engineering and Information Technology, University of Naples “Federico II”, 80125 Naples, Italy; carloricciardi.93@gmail.com; 2 Bioengineering Unit, Institute of Care and Scientific Research Maugeri, 27100 Pavia, Italy; 3 Maxillofacial Surgery Unit, Department of Neurosciences, Reproductive and Odontostomatological Sciences, University Hospital of Naples “Federico II”, 80131 Napoli, Italy; giovanni.dellaversanaorabona@unina.it (G.D.O.); alfonsosorrentino87@hotmail.it (A.S.); 4 Department of Advanced Biomedical Sciences, University Hospital of Naples “Federico II”, 80131 Naples, Italy; ila.picone@hotmail.it (I.P.); antonellafiorillo@virgilio.it (A.F.); 5 Department of Public Health, University Hospital of Naples “Federico II”, 80131 Naples, Italy; immalatessa@gmail.com (I.L.); triassi@unina.it (M.T.); 6 Interdepartmental Center for Research in Healthcare Management and Innovation in Healthcare (CIRMIS), University of Naples “Federico II”, 80131 Naples, Italy

**Keywords:** health technology assessment, six sigma, maxillofacial surgery, healthcare, drugs

## Abstract

Squamous cell carcinoma represents the most common cancer affecting the oral cavity. At the University of Naples “Federico II”, two different antibiotic protocols were used in patients undergoing oral mucosa cancer surgery from 2006 to 2018. From 2011, there was a shift; the combination of Cefazolin plus Clindamycin as a postoperative prophylactic protocol was chosen. In this paper, a health technology assessment (HTA) is performed by using the Six Sigma and DMAIC (Define, Measure, Analyse, Improve, Control) cycle in order to compare the performance of the antibiotic protocols according to the length of hospital stay (LOS). The data (13 variables) of two groups were collected and analysed; overall, 136 patients were involved. The American Society of Anaesthesiologist score, use of lymphadenectomy or tracheotomy and the presence of infections influenced LOS significantly (*p*-value < 0.05) in both groups. Then, the groups were compared: the overall difference between LOS of the groups was not statistically significant, but some insights were provided by comparing the LOS of the groups according to each variable. In conclusion, in light of the insights provided by this study regarding the comparison of two antibiotic protocols, the utilization of DMAIC cycle and Six Sigma tools to perform HTA studies could be considered in future research.

## 1. Introduction

Cancer of the oral cavity is one of the most common malignant neoplasms worldwide [1] and can affect all types of tissues that compose the mouth, including muscles, bones, salivary gland and mucosa. Among these malignancies, squamous cell carcinoma (SCC) represents the most common cancer affecting the oral cavity [2] and is the sixth most common cancer [3].

Multiple variables influence the incidence of oral cancer, including poor oral hygiene, smoking [4], alcohol habit, viral infections and chronic inflammation [5,6]. SCC of oral mucosa can also arise without any previous risk factors [7]. Despite the fact that it is easy and requires clinical inspection and a biopsy of the neoplasm, representing the gold standard procedure [8], oral cancer diagnosis is often discovered only when patients present an advanced stage of disease. The treatment of oral cancer requires different approaches, such as chemotherapy and radiotherapy, but surgery remains the main treatment [2].

This study is based on the analysis of clinical outcomes of patients treated for oral cancer involving oral mucosa, excluding other cancers deriving from the tongue, gum, muscles and bones. In our study, we considered all surgeries performed with the removal of oral mucosa with or without reconstruction. The surgical management of oral cancer is complex because of different aspects. First, the mouth is involved in various important physiological functions, such as speech breathing, deglutition and mastication [9], but it is also important for its aesthetic reasons. It presents intrinsic difficulties due to the presence of oral bacterial flora and some physic characteristic, such as humidity, heat and movement [10,11]. Reconstructive surgery is required to restore the anatomy and/or the functionality of the mouth or to reduce oral disabilities after big tissue removals.

Regarding the operations involving removal and reconstruction of oral mucosa, surgical options preview primary closure or the use of local or free flaps. Depending on tumor stage and lymph nodes involvement, some more surgical procedure may be considered, such as neck dissection and tracheotomy. Neck dissection consists in the removal of lymph nodes of the neck to prevent metastatic evolution. The need of prophylactic neck dissection where there is no evidence of cervical lymph nodes involvement is still under discussion [12]. Despite the high morbidity of this surgical procedure, the higher costs and the longer time of postoperative hospitalization, many studies have evidenced that prophylactic neck dissection seems to be related to better survival rates [13,14]. Tracheotomy is required when big tissue removal can result in an increased risk of edema, hematoma and hemorrhage that can cause a partial or total airway occlusion.

At the Department of Maxillofacial Surgery of the University of Naples “Federico II”, two different antibiotic protocols were used in patients undergoing oral mucosa cancer surgery from 2006 to 2018, according to internal guidelines. A postoperative antibiotic protocol with Ceftriaxone was used for patients without allergy from 2006 to 2018. From 2011, there was a shift to the use of the combination of Cefazolin plus Clindamycin as a postoperative prophylactic protocol.

The aim of this work was to perform a health technology assessment (HTA) in order to compare the performance of the above-mentioned antibiotics by studying the postoperative length of stay (LOS), measured in days. The Six Sigma (SS), by using the DMAIC (Define-Measure-Analyse-Improve-Control) cycle, was used as a tool of the HTA: the influence of some variables on the postoperative LOS was analysed, and patients’ hospitalization time from surgery until discharge was the focus.

### Literature Review: Health Technology Assessment and Six Sigma

SS is an innovative quality management method that focuses on reducing variations, measuring defects and improving the quality of products, processes and services. It was first introduced by Bill Smith to the Motorola Corporation in the 1980s and further developed by General Electric in the late 1990s [15].

DMAIC structure is one of the quality improvement methods used in the SS concept considered by many professionals to be the main reason for its success:

- The Define phase identifies the project, the problem and the objective.
- In the Measure phase, the current process that needs improvement is quantitatively described.
- In the Analyse phase, the statistical analysis is used to understand causes and effects in relation to the current process.
- The Improve phase allows users to develop a plan that can be validated by statistical data to improve the process. In this research, this phase will be used to compare the two analysed approaches.
- The Control phase establishes a monitoring tool or mechanisms to ensure that the process is supported and to design effective quality controls [16,17]; in this research, this phase will be used to evaluate and compare the two analysed approaches.

Research studies in the healthcare sector confirm the validity of the methodology and several studies in literature discussed the administration of drugs analysed through DMAIC cycle.

Yousef N. and Yousef F. studied the medication use process by using the DMAIC approach in order to find out the real reasons behind drug administration errors with the aim of reducing errors to 1% [17]. Other authors used SS methodology to improve time to antibiotics for children with chemotherapy-induced febrile neutropenia presenting to the emergency department: indeed, SS methodology effectively identified barriers and provided solutions to remove them [18,19].

The purpose of the study carried out Downen J. and Jaeger C. was to decrease the rate of missed intravenous to oral medication conversion opportunities for eligible patients, which aligns with the strategic plan for quality improvement of resource utilization and reduces delays when using the LSS approach and DMAIC [20].

Today, healthcare is constantly evolving, and it seeks to offer improvements in technology and patient treatment. It is a complex network in which resource limitations, errors and complications threaten the security of patient care and service provision [21]. Therefore, the use of management methods and tools for the quality control of health services is necessary [22,23,24]: several decision making strategies, software and simulation approaches and modern quality management tools, such as the SS concept, can offer realistic answers to reach higher levels of excellence in the health context by helping the assessment of technologies with HTA studies [25,26,27,28], elaboration and simulation of complex data [29,30,31,32] and the implementation of machine learning algorithms [33,34,35,36].

A methodology based on reducing variations, measuring defects and improving the quality of products, processes and services turns out to be SS. It has been recognized globally in the service sector, and the use and the success of the SS in healthcare in the last decade have been very significant as a practice of continuous improvement [37]. SS projects use a DMAIC structure to improve processes [38].

However, this methodology, as emerged from scientific studies, is often associated with lean thinking. The combination of these two methodologies aims to improve services to meet customer needs by eliminating waste and reducing costs [39,40]. Recently, SS has been used with Agile methodology in an Italian hospital context [41].

HTA has been used in combination with other methods as well; several studies performed HTA by using techniques and methodologies such as analytical hierarchical process, Likert scale, modelling and simulations [25,26,27].

The SS framework has not been formally applied to the comparison of the performance of two antibiotic protocols until last year: For the first time, SS has been used as a tool of HTA to compare two drugs recently with good results [42,43]. Similarly, it has not only been employed for comparing cemented and uncemented prostheses in orthopaedics [44], two surgical approaches for abdominoplasty or two prostheses for immediate breast reconstruction in plastic surgery [45,46], but it was also combined with regression analyses more recently in maxillofacial surgery [47]. All these applications demonstrate the feasibility of using Six Sigma for performing HTA studies, but this study specifically investigates patients with cancer in the mucosa tissue, which has not been analysed before by using this approach.

## 2. Methods

### 2.1. Context

All data analysed in this study were based on surgeries performed at the Maxillofacial Surgery Unit of the University Hospital of Naples “Federico II”. The Unit is divided in two different floors. At the ground floor, there is the day hospital part of the ward with two desks and the possibility to visit two different patients at the same time, the day surgery ambulatory where minor surgeries are performed daily, the direction and some medical offices. The hospital ward, the operating rooms, the nursery, the pharmacy and some rooms for the medical and nursing teams are located on the first floor. The Maxillofacial Surgery Unit of “Federico II” consists also in an acceptance office, a library and the residency office. In the hospital ward, there are 9 rooms with 22 beds for the patients and some rooms for surgeons and nurses. Near the hospital ward, there are two operating rooms that work at the same time.

### 2.2. Collection of Data

In this study, two groups of patients were analysed, and the data were collected from the Department of Maxillofacial Surgery of the University of Naples “Federico II”. The first one was treated with Cefazolin plus Clindamycin from 2011 to 2018, while the second one was treated with Ceftriaxone since 2006 to 2017. The first group includes 51 patients, while the second one includes 85 patients. The data were collected from medical records, and statistical tests were performed by using IBM SPSS Statistics version 25 (United States). The following exclusion criteria were applied:

- Patients with postoperative LOS ≤ 2 because the target of the study was ordinary hospitalization.
- Patients who underwent an antibiotic shift during their hospital stay (8 treated with Ceftriaxone and 15 with Cefazolin plus Clindamycin).
- Patients with missing data because they could have compromised the result of the analyses.

All patients were included without exclusion due to clinical history.

Each patient was analysed according to the following variables:

- Gender;
- Age;
- American Society of Anaesthesiologists (ASA) score;
- Oral hygiene;
- Diabetes;
- Cardiovascular diseases;
- Surgical intervention;
- Flap;
- Lymphadenectomy;
- Tracheotomy;
- Surgical site infections;
- Dehiscence;
- Fistulae.

### 2.3. Define

**Figure 1:**
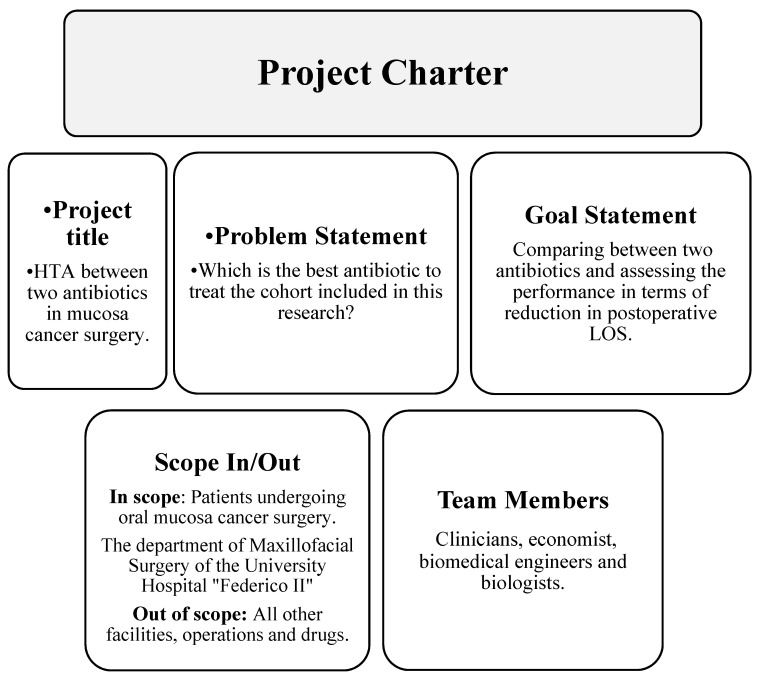
Project charter regarding the main aspects of the research.

The aim of the Define phase is to determine a working group and to divide tasks for analysis. The multidisciplinary team is composed of clinicians, an economist, biomedical engineers and two biologists with experience in health management. The team was responsible for collecting data from patients with oral cancer from medical records and consecutive data analyses considering the influence of some variables. The leader supervised and coordinated the study and interpretation of the data. The critical quality of the research was the LOS. A project charter was created (Figure 1) to show the main aspects of the research.

Then, a SIPOC (Suppliers-Inputs-Processes-Output-Customers) diagram was designed by the team to show the main actors of the projects. This tool is very useful for identifying all relevant elements of a process improvement project before the work begins (Table 1).

### 2.4. Measure

The purpose of this phase is to measure the performance of the antibiotics studied.

The dataset is made up of two groups of patients: The first includes 51 patients treated with Cefazolin plus Clindamycin, while the second includes 85 patients treated with Ceftriaxone. The time frame in which they were collected is from 2011 to 2018 for patients treated with Cefazolin plus Clindamycin and from 2006 to 2018 for those treated with Ceftriaxone. All the collected variables were analysed, and patients undergoing an antibiotic shift during their hospitalization or had missing data were not included in the analysis because they could have compromised the results.

**Figure 2:**
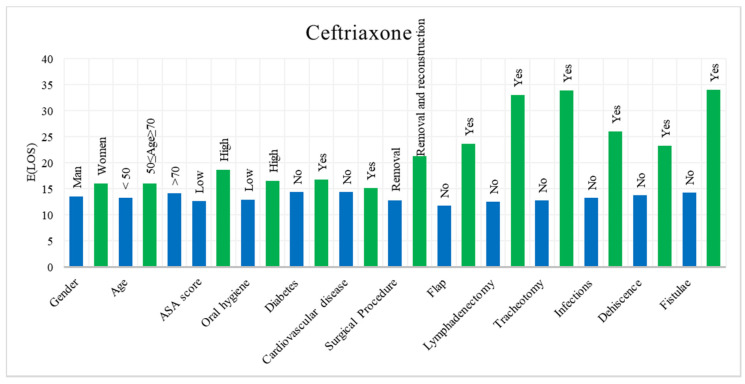
Mean LOS for each category of variables regarding Ceftriaxone.

**Figure 3:**
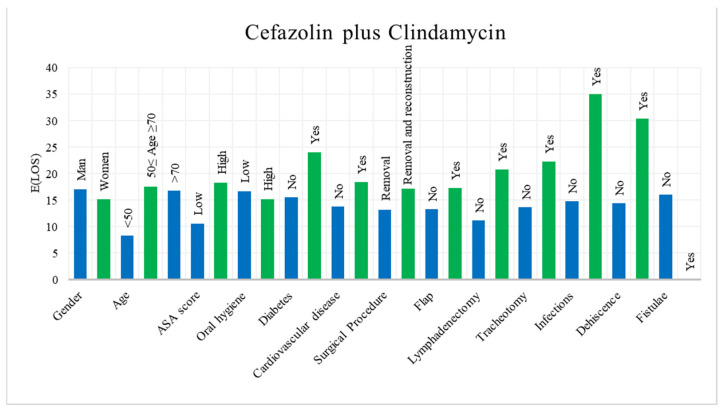
Mean LOS for each category of variables regarding Cefazolin plus Clindamycin.

In the descriptive analysis, the postoperative LOS of both antibiotics was reported according to each variable. The results show that, in patients treated with Cefazolin plus Clindamycin, the mean LOS is 1600 days with a standard deviation of 982 days, whereas for Ceftriaxone the mean is 1471 days with a standard deviation of 1147 days. Figure 2 shows a histogram with the mean postoperative LOS of patients treated with Ceftriaxone, while in Figure 3 the histogram of those treated with Cefazolin plus Clindamycin is displayed.

### 2.5. Analyse

In this phase, the data were analysed in order to evaluate which variables can influence postoperative LOS. First, the Kolmogorov–Smirnov test was used in order to evaluate the data distribution.

Patients treated with Ceftriaxone showed a non-normal trend with a *p*-value < 0.001; thus, the Mann–Whitney U test was used to study dichotomous variables and Kruskal–Wallis was used for non-dichotomous variables (age). The normality Kolmogorov–Smirnov test on patients treated with Cefazolin plus Clindamycin distinctly showed a *p*-value 0.196 (normal distribution); therefore, *t*-test and ANOVA (only for age) were employed.

The results regarding Ceftriaxone are shown in Table 2. Several significances were found: a high ASA, both removal and reconstruction surgeries score, using flap, lymphadenectomy and tracheotomy and the presence of infections and fistulae caused the LOS to increase significantly.

As shown below in Table 3, several variables influence postoperative LOS of patients treated with Cefazolin plus Clindamycin: a high ASA, using lymphadenectomy and tracheotomy, the presence of infections and dehiscence score caused the LOS to increase significantly.

The workflow of the activities carried on in the ward has already been shown in previous research [42]; the activities include the following: arrival of the patient, prehospitalization (or preoperative activities when the patient is not prehospitalized), surgical actives and postoperative activities until discharge.

### 2.6. Improve

To date, a correct antibiotic prophylactic protocol for head and neck cancer surgery has not been clearly defined yet [48].

We divided our population of patients into two groups depending on the use of Ceftriaxon, a third-generation cephalosporin, and on the use of the association of Cefazolin and Clindamycin, our second antibiotic protocol.

Ceftriaxone is a third-generation cephalosporin antibiotic belonging to the beta lactam family. It operates by inhibiting the synthesis of the bacterial cell wall. For this reason, antibiotics belonging to the class of cephalosporins are bactericides. In clinical practice, Ceftriaxon is generally used to treat most of the infections sustained by antibiotic resistant bacteria. It is active on Gram-positive bacteria with a tropism to skin and soft tissues infection because of its action on the bacterial cell wall. Due to the increased risk of antibiotic resistance from bacteria, Ceftriaxon should not be used as a first-choice antibiotic, despite the fact that it represents the main choice for empiric antibiotic therapy due to its wide spectrum antibiotic coverage.

Cefazolin is a semi-synthetic beta-lactam antibiotic belonging to first-generation cephalosporins. This antibiotic is active against a large population of bacteria, including Methicillin-sensible Staphylococcus Aureus, Streptocococcus Pneumoniae, Clostridium perfringens and Lysteria monocytogenes. It is less active on Gram-negative bacteria and not active against viruses. As antibiotic protocol in maxillofacial surgery, Cefazolin is used for infections that affect the upper respiratory and the upper aerodigestive tracts and soft tissues.

Clindamycin is an antibiotic belonging to the lincosamide class that acts with a bacteriostatic mechanism by interfering in the replication of different bacteria. Clindamycin act in inhibiting the synthesis of the proteins of the bacterial cells. It is used against the infections sustained by anaerobic bacteria and Methicillin-resistant Staphylococcous Aureus. In clinical practice, it is used for bacterial infections involving bones, oral cavity, teeth, upper respiratory and digestive tracts. It is active also on soft tissue, especially to treat infections affecting the oral floor and the neck. Clindamycin is also used as an alternative when the patient is allergic to beta lactam antibiotics.

In the Maxillofacial Unit of University Hospital “Federico II”, an association of Cefazolin and Clindamycin has been used since 2011 as an antibiotic postoperative protocol against the main classes of bacteria affecting the head and neck district. The sections may be divided by subheadings. It should provide a concise and precise description of the experimental results, their interpretation and the experimental conclusions that can be drawn.

### 2.7. Control

**Figure 4:**
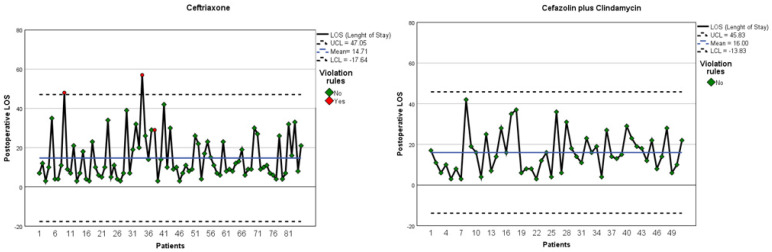
Control Chart before (**left**) and after (**right**) the new postoperative antibiotic protocol.

**Figure 5:**
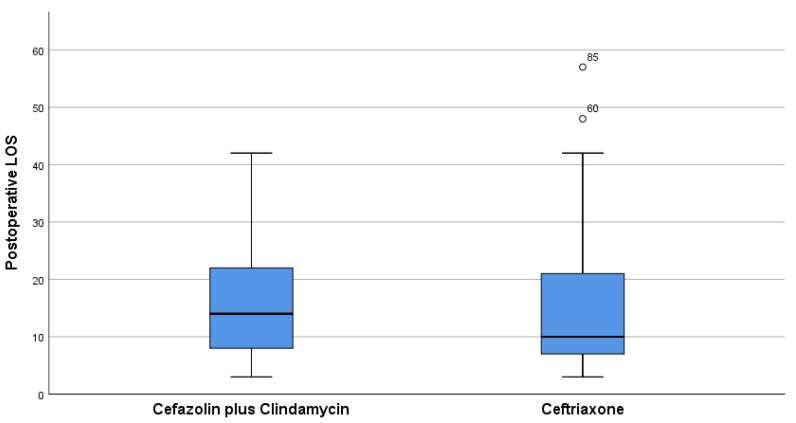
As per Six Sigma methodology, visual management (Box plot) is employed to provide readers with a graphical representation of LOS for both groups. The numbers over the boxes can be considered outliers.

Control charts were used to monitor the performance of the key variable. After implementing the actions described in the improve phase, a decrease in the mean of the postoperative LOS of the patients treated with Ceftriaxone was observed, as shown in the control charts before and after the implementation of the new postoperative antibiotic protocol with Cefazolin plus Clindamycin in Figure 4. The lower and upper control limits of the individual values showed a reduction, indicating more stable administration of Cefazolin plus Clindamycin. A box plot was developed and shown in Figure 5, which clearly highlights the decrease in the mean in the Ceftriaxone group of LOS measured in days. In Table 4, the percentage difference of the mean of postoperative LOS was calculated and reported. The mean of patients treated with Ceftriaxone had a decreasing percentage of −8.1%.

A control plan was established to ensure continuous improvements in the future in terms of the performance of both antibiotics:

- Following the guidelines to improve administration, drawn up according to the influence of clinical characteristics and complications, as from the analyses carried out in this study;
- Periodic review meetings to evaluate the maxillofacial surgery process;
- Internal audit and production of reports that highlight the trend of patients’ LOS measured in days.

## 3. Results

The Kolmogorov–Smirnov test for the normality of data showed a *p*-value lower than 0.01; thus, each category was studied by using non-parametric statistical tests. Dichotomous groups were analysed with a Mann–Whitney U test, while groups with more than two categories (only age) were analysed with a Kruskal–Wallis test. The percentage difference in the average of the postoperative LOS is calculated between the initial value of Cefazolin plus Clindamycin and the final value Ceftriaxone. Table 4 shows all the results. The test on all patients showed that the difference in antibiotic performance, in terms of postoperative LOS, is not statistically significant (*p*-value 0.197). With respect to the categories of the variables, patients with lymphadenectomy (*p*-value 0.023) and tracheotomy (*p*-value 0.050) treated with Cefazolin plus Clindamycin have, on average, a lower postoperative LOS in a statistically significant manner. The percentage differences explain that, on average, postoperative LOS does not follow a trend line in favour of a single antibiotic. The most relevant percentage differences indicate that patients under the age of 50 treated with Ceftriaxone had an increase in LOS of 60.1% compared to those treated with Cefazolin plus Clindamycinl; on the contrary, patients with diabetes treated with Cefazolin plus Clindamycin had a decrease in LOS of −30.3%.

The chi-square test was conducted for a population demographic study; the results are shown in Table 5. Only some variables showed a statistically significant difference in frequency between the two groups of patients. However, the decrease in the number of patients treated with Ceftriaxone is statistically significant in the following cases: patients’ frequencies with a low ASA score; receiving a surgical procedure of removal plus reconstruction; a flap, a lymphadenectomy or a tracheotomy are greater in the Cefazolin plus Clindamycin group.

## 4. Discussion

At the Department of Maxillofacial Surgery of the University of Naples “Federico II”, a comparison study was carried out between two antibiotics Ceftriaxone and Cefazolin plus Clindamycin, investigating the problem of prolonging postoperative LOS in patients undergoing oral mucosa cancer surgery.

As shown by previous studies, the SS methodology was found to be useful for improving decision making in healthcare [49]. In particular, the application of this methodology often seems to result in statistically significant reduction in LOS [17,39,40,42]. Therefore, SS methodology was applied to evaluate antibiotic performance and reduce postoperative LOS. The DMAIC cycle was carried out, and various SS tools were used for a clear visualization of the project: representative graphs (SIPOC diagram, histograms, control charts and box plot) and tables summarizing the statistical tests (Mann–Whitney U, Kruskal–Wallis, *t*-test and ANOVA).

From the analysis in Table 2 and Table 3, some general conclusions can be synthetized. The ASA classification system is a method for characterizing patient’s operative risk: We observed that high ASA scores significantly increase hospitalization. This can be explained because a high ASA score is assigned to patients with an increased anesthetic risk, indicating a major risk of developing complications [50,51]. Patients with associated pathologies, such as cardiovascular disease and diabetes, need different preoperative and postoperative management with respect to healthy people that undergo surgery. This increases the time of hospitalization because of the need of a multidisciplinary approach towards patients.

Depending on the type of surgery, we observed that the need of reconstruction, especially when the use of a flap is required, causes an increase in the incidence of surgical site infections and, consequently, LOS [52]. A simple removal without reconstruction predisposes the patient to different outcomes: A minor removal of tissue often results in quick postoperative LOS because of the better healing process; when increased removal is performed, we can observe a major incidence of postoperative morbidity due to a bigger exposition of surgical surfaces and an increased incidence of physiological and anatomical deficits. The need of reconstruction results in an increased time period of hospitalization. To reconstruct a surgical site, surgeons need to use local tissues or pedicled/free flaps. The use of these techniques causes major exposition of the body to surgical procedure, with increased morbidity [53]. The use of pedicled or free flaps also results in increased risk of surgical site infections due to the contamination of surgical wound with bacteria coming from other parts of the body. Tracheotomy and neck dissection cause an increased LOS because of the same reason previously described about surgical gaps and tissue exposure. A major surface involved in the surgery will inevitably tend to develop complications. It is well-known that skin represents a barrier to external factors, including bacteria. When performing tracheotomy or a neck dissection, skin incision with exposure of the surgical site to external factors is inevitable and cannot be avoided. Among these factors that can determine complications in oral cancer surgery, we can list bacteria coming from the external environment or different part of the body and other components coming from the same patient, such as blood, saliva and food [10,54,55]. These factors influence postoperative LOS because they can cause dehiscence, fistulae and surgical site infections (SSIs). In our analysis, we observed no significant difference between the two antibiotic protocols to prevent SSIs in oral cancer surgery related to mucosa and to reduce LOS. This can be explained because both antibiotics are active on soft tissues.

Other factors that seem to reduce LOS when referred to the Cefazolin/Clindamycin protocols are a low ASA score and an age lower than 50 years. Reduced times of hospitalization for patients with low ASA scores and age lower than 50 can be explained because young patients present less comorbidities in comparison to older people. Young people are generally healthier and present better healing process. They are also more compliant to therapies, and their health status can be better managed. Older people have a major incidence of comorbidities because the senescence process determines body weakness and the appearance of chronicle pathologies that need to be considered for their management.

Moreover, from Table 4, we can observe an increased percentage of better outcomes when we consider surgical procedure. Cefazolin/Clindamycin protocol seems to reduce LOS in comparison with Ceftriaxone when flaps are used for reconstruction (+36.8%). Without considering the high incidence of antibiotic resistance, the reduced time of hospitalization can be explained in this case with a major covering on bacterial population due to the use of antibiotic association with respect to a single antibiotic protocol.

The other facets of HTA need to be discussed too: the two drugs are equally approved, have similar costs and are both currently used in clinical practice without requiring different organizational pathways. Therefore, it appears that the two antibiotic protocols are almost equivalent with respect to the number of complications (infections, dehiscence and fistulae) and the LOS.

## 5. Conclusions

In conclusion, this study proved the utility of applying SS methodology using the DMAIC cycle for comparing the performance of the two antibiotics administered after surgery for cancer of the oral mucosa. Obviously, SS is a management and non-medical tool from the beginning; however, it could be a good method for analysing clinical and surgical variables to support the decision-making process of the doctors.

## Figures and Tables

**Table 1:** SIPOC diagram.

Supplier	Inputs	Process		Outputs	Customers
University Hospital of Naples “Federico II”	Needs of patients	Arrival at the hospital	Surgery	Shorter recovery	Patients
Clinical staff	Maxillofacial surgery	Recovery	Postoperative activities	Improved outcome of patients	University Hospital of Naples “Federico II”
		Preoperative activities	Discharge	Ensuring fewer complications	

**Table 2:** Variables influencing LOS for Ceftriaxone have been studied by comparing all the categories related to each variable by applying Mann–Whitney U/Kruskal–Wallis tests. * Significance at 0.05; ** significance at 0.01; *** significance at 0.001.

Variable	Category	LOS (Mean ± dev std)	*n*	*p*-Value
Gender	Men	13.48 ± 10.77	44	0.441
	Women	16.02 ± 12.17	41	
Age	<50	13.27 ± 12.80	15	0.640
	50 ≤ Age ≤ 70	16.00 ± 13.52	34	
	>70	14.08 ± 8.65	36	
ASA score	Low	12.60 ± 10.28	55	0.008 **
	High	18.57 ± 12.67	30	
Oral hygiene	Low	12.93 ± 9.42	43	0.305
	High	16.52 ± 13.11	42	
Diabetes	No	14.41 ± 11.63	74	0.355
	Yes	16.73 ± 10.63	11	
Cardiovascular disease	No	14.42 ± 9.89	50	0.601
	Yes	15.11 ± 13.56	35	
Surgical Procedure	Removal	12.82 ± 10.27	66	0.003 **
	Removal and reconstruction	21.26 ± 13.22	19	
Flap	No	11.78 ± 8.30	64	<0.001 ***
	Yes	23.62 ± 14.99	21	
Lymphadenectomy	No	12.54 ± 8.96	76	<0.001 ***
	Yes	33.00 ± 14.44	9	
Tracheotomy	No	12.71 ± 8.98	77	0.001 ***
	Yes	33.88 ± 15.51	8	
Infections	No	13.20 ± 10.38	75	0.005 **
	Yes	26.00 ± 13.47	10	
Dehiscence	No	13.70 ± 10.29	76	0.061
	Yes	23.22 ±17.23	9	
Fistulae	No	14.24 ±11.20	83	0.017 *
	Yes	34.00 ± 1.41	2	

**Table 3:** Variables influencing LOS for Cefazolin plus Clindamycin have been studied by comparing all the categories related to each variable by applying the Mann–Whitney U/Kruskal–Wallis tests. * Significance at 0.05; ** significance at 0.01; *** significance at 0.001.

Variable	Category	LOS (Mean ± dev std)	*n*	*p*-Value
Gender	Men	17.00 ± 11.40	23	0.515
	Women	15.18 ± 8.82	28	
Age	<50	8.29 ± 4.96	7	0.585
	50 ≤ Age ≤ 70	17.57 ± 11.18	27	
	>70	16.71 ± 7.66	17	
ASA score	Low	10.53 ± 9.59	15	0.009 **
	High	18.28 ± 9.11	36	
Oral hygiene	Low	16.63 ± 10.31	30	0.587
	High	15.10 ± 9.25	21	
Diabetes	No	15.50 ± 9.37	48	0.148
	Yes	24.00 ± 15.72	3	
Cardiovascular disease	No	13.77 ± 8.22	26	0.099
	Yes	18.32 ± 10.94	25	
Surgical Procedure	Removal	13.20 ± 8.21	15	0.192
	Removal and reconstruction	17.17 ± 10.30	36	
Flap	No	13.25 ± 7.83	16	0.179
	Yes	17.26 ± 10.47	35	
Lymphadenectomy	No	11.12 ± 6.75	25	<0.001 ***
	Yes	20.69 ± 10.12	26	
Tracheotomy	No	13.65 ± 9.10	37	0.004 **
	Yes	22.21 ± 9.19	14	
Infections	No	14.81 ± 8.72	48	<0.001 ***
	Yes	35.00 ± 7.00	3	
Dehiscence	No	14.43 ± 8.71	46	<0.001 ***
	Yes	30.40 ± 8.08	5	
Fistulae	No	16.00 ± 9.82	51	N.A.
	Yes	N.A.	0	

**Table 4:** Statistical analysis of LOS: a comparison between Ceftriaxone and Cefazolin plus Clindamycin is performed for the categories of each variable. * Significance at 0.05; ** significance at 0.01; *** significance at 0.001.

Variables	Category	Ceftriaxone (Mean ± dev std)	Cefazolin Plus Clindamycin (Mean ± dev std)	Difference of the Mean (%)	*p*-Value
All patients		14.71 ± 11.47	16.00 ± 9.82	−8.1%	0.197
Gender	Men	13.48 ± 10.77	17.00 ± 11.40	−20.7%	0.236
	Women	16.02 ± 12.17	15.18 ± 8.82	5.5%	0.732
Age	<50	13.27 ± 12.80	8.29 ± 4.96	60.1%	0.490
	50 ≤ Age ≤ 70	16.00 ± 13.52	17.57 ± 11.18	−8.9%	0.299
	>70	14.08 ± 8.65	16.71 ± 7.66	−15.7%	0.185
ASA score	Low	12.60 ± 10.28	10.53 ± 9.59	19.7%	0.615
	High	18.57 ± 12.67	18.28 ± 9.11	1.6%	0.671
Oral hygiene	Low	12.93 ± 9.42	16.63 ± 10.31	−22.2%	0.078
	High	16.52 ± 13.11	15.10 ± 9.25	9.4%	0.907
Diabetes	No	14.41 ± 11.63	15.50 ± 9.37	−7.0%	0.175
	Yes	16.73 ±10.63	24.00 ± 15.72	−30.3%	0.456
Cardiovascular disease	No	14.42 ± 9.89	13.77 ± 8.22	4.7%	0.969
	Yes	15.11 ± 13.56	18.32 ± 10.94	−17.5%	0.086
Surgical Procedure	Removal	12.82 ± 10.27	13.20 ± 8.21	−2.9%	0.630
	Removal and reconstruction	21.26 ±13.22	17.17 ± 10.30	23.8%	0.357
Flap	No	11.78 ± 8.30	13.25 ± 7.83	−11.1%	0.344
	Yes	23.62 ± 14.99	17.26 ± 10.47	36.8%	0.165
Lymphadenectomy	No	12.54 ± 8.96	11.12 ± 6.75	12.8%	0.714
	Yes	33.00 ±14.44	20.69 ± 10.12	59.5%	0.023 *
Tracheotomy	No	12.71 ± 8.98	13.65 ± 9.10	−6.9%	0.471
	Yes	33.88 ±15.51	22.21 ± 9.19	52.5%	0.050 *
Infections	No	13.20 ± 10.38	14.81 ± 8.72	−10.9%	0.114
	Yes	26.00 ±13.47	35.00 ± 7.00	−25.7%	0.287
Dehiscence	No	13.70 ± 10.29	14.43 ± 8.71	−5.1%	0.317
	Yes	23.22 ±17.23	30.40 ± 8.08	−23.6%	0.298
Fistulae	No	14.24 ±11.20	16.00 ± 9.82	−11.0%	0.130
	Yes	34.00 ± 1.41	0	N.A.	

N.A. = Not Applicable because the sample size does not allow comparison.

**Table 5:** A Demographic study is performed by using the chi-square test to investigate the frequencies related to each variable. * Significance at 0.05; ** significance at 0.01; *** significance at 0.001.

Variables	Category	Ceftriaxone (*n*)	Cefazolin Plus Clindamycin (*n*)	*p*-Value
Gender	Men	44	23	0.452
	Women	41	28	
Age	<50	15	7	0.340
	50 ≤ Age ≤ 70	34	27	
	>70	36	17	
ASA score	Low	55	15	<0.001 ***
	High	30	36	
Oral hygiene	Low	43	30	0.351
	High	42	21	
Diabetes	No	74	48	0.190
	Yes	11	3	
Cardiovascular disease	No	50	26	0.372
	Yes	35	25	
Surgical Procedure	Removal	66	15	<0.001 ***
	Removal and reconstruction	19	36	
Flap	No	64	16	<0.001 ***
	Yes	21	35	
Lymphadenectomy	No	76	25	<0,001 ***
	Yes	9	26	
Tracheotomy	No	77	37	0.006 **
	Yes	8	14	
Infections	No	75	48	0.259
	Yes	10	3	
Dehiscence	No	76	46	0.884
	Yes	9	5	
Fistulae	No	83	51	0.270
	Yes	2	0	

## Data Availability

The data presented in this study are available on request from the corresponding author.

## Abbreviations

ASA: American Society of Anesthesiologists. DMAIC: Define, Measure, Analyse, Improve and Control. LOS: Length of Hospital Stay. LSS: Lean Six Sigma. SIPOC: Suppliers, Inputs, Processes, Output and Customers. SCC: Squamous cell carcinoma. SS: Six Sigma. SSIs: Surgical Site Infections.

## References

[1] Montero P.H., Patel S.G. (2015). Cancer of the Oral Cavity. Surg. Oncol. Clin. N. Am..

[2] Ettinger K.S., Ganry L., Fernandes R.P. (2019). Oral Cavity Cancer. Oral. Maxillofac. Surg. Clin. N. Am..

[3] Tsantoulis P.K., Kastrinakis N.G., Tourvas A.D., Laskaris G., Gorgoulis V.G. (2007). Advances in the biology of oral cancer. Oral Oncol..

[4] Chaturvedi P., Singh A., Chien C.Y., Warnakulasuriya S. (2019). Tobacco related Oral Cancer. BMJ.

[5] Gillison M.L., Chaturvedi A.K., Anderson W.F., Fakhry C. (2015). Epidemiology of human papillomavirus-positive head and neck squamous cell carcinoma. J. Clin. Oncol..

[6] Yete S., D’Souza W., Saranath D. (2018). High-Risk Human Papillomavirus in Oral Cancer: Clinical Implications. Oncology.

[7] Yang E.C., Tan M.T., Schwarz R.A., Richards-Kortum R.R., Gillenwater A.M., Vigneswaran N. (2018). Noninvasive diagnostic adjuncts for the evaluation of potentially premalignant oral epithelial lesions: Current limitations and future directions. Oral Surg. Oral Med. Oral Pathol. Oral Radiol..

[8] Porter S., Gueiros L.A., Leão J.C., Fedele S. (2018). Risk factors and etiopathogenesis of potentially premalignant oral epithelial lesions. Oral Surg. Oral Med. Oral Pathol. Oral Radiol..

[9] Wong T., Wiesenfeld D. (2018). Oral Cancer. Aust. Dent. J..

[10] Durand M.L., Yarlagadda B.B., Rich D.L., Lin D.T., Emerick K.S., Rocco J.W., Deschler D.G. (2015). The time course and microbiology of surgical site infections after head and neck free flap surgery. Laryngoscope.

[11] Avery C.M., Ameerally P., Castling B., Swann R.A. (2006). Infection of surgical wounds in the maxillofacial region and free flap donor sites with methicillin-resistant Staphylococcus aureus. Br. J. Oral Maxillofac. Surg..

[12] De Bree R., Takes R.P., Shah J.P., Hamoir M., Kowalski L.P., Robbins K.T., Rodrigo J.P., Sanabria A., Medina J.E., Rinaldo A. (2019). Elective neck dissection in oral squamous cell carcinoma: Past, present and future. Oral Oncol..

[13] D’Cruz A.K., Vaish R., Kapre N., Dandekar M., Gupta S., Hawaldar R., Agarwal J.P., Pantvaidya G., Chaukar D., Deshmukh A. (2015). Elective versus Therapeutic Neck Dissection in Node-Negative Oral Cancer. N. Engl. J. Med..

[14] Huang S.F., Chang J.T., Liao C.T., Kang C.J., Lin C.Y., Fan K.H., Wang H.M., Chen I.H. (2015). The role of elective neck dissection in early stage buccal cancer. Laryngoscope.

[15] De Koning H., de Mast J. (2006). A Rational Reconstruction of Six Sigma’s Breakthrough Cookbook. Int. J. Qual. Reliab. Manag..

[16] Yousef N., Yousef F. (2017). Using total quality management approach to improve patient safety by preventing medication error incidences. BMC Health Serv. Res..

[17] Ricciardi C., Fiorillo A., Valente A.S., Borrelli A., Verdoliva C., Triassi M., Improta G. (2019). Lean Six Sigma approach to reduce LOS through a diagnostic-therapeutic-assistance path at A.O.R.N.A. Cardarelli. TQM J..

[18] Geerlinks A.V., Digout C., Bernstein M., Chan A., MacPhee S., Pambrun C., Gallant G., Wyatt L., Fernandez C.V., Price V.E. (2020). Improving Time to Antibiotics for Pediatric Oncology Patients With Fever and Suspected Neutropenia by Applying Lean Principles. Pediatr. Emerg. Care.

[19] Maniscalco G.T., Cerillo I., Servillo G., Napolitano M., Guarcello G., Abate V., Improta G., Florio C. (2018). Early neutropenia with thrombocytopenia following alemtuzumab treatment for multiple sclerosis: Case report and review of literature. Clin. Neurol. Neurosurg..

[20] Downen J., Jaeger C. (2020). Quality improvement of intravenous to oral medication conversion using Lean Six Sigma methodologies. BMJ Open Qual..

[21] Udayai K., Kumar P. (2012). Implementing Six Sigma to improve hospital discharge process. Int. J. Pharm. Sci. Res..

[22] Akifuddin S., Khatoon F. (2015). Reduction of Complications of Local Anaesthesia in Dental Healthcare Setups by Application of the Six Sigma Methodology: A Statistical Quality Improvement Technique. J. Clin. Diagn. Res..

[23] Improta G., Mazzella V., Vecchione D., Santini S., Triassi M. (2019). Fuzzy logic–based clinical decision support system for the evaluation of renal function in post-Transplant Patients. J. Eval. Clin. Pract..

[24] Cortesi P.A., Castaman G., Trifiro G., Creazzola S.S., Improta G., Mazzaglia G., Molinari A.C., Mantovani L.G. (2020). Cost-Effectiveness and Budget Impact of Emicizumab Prophylaxis in Haemophilia A Patients with Inhibitors. Thromb. Haemost..

[25] Improta G., Perrone A., Russo M.A., Triassi M. (2019). Health technology assessment (HTA) of optoelectronic biosensors for oncology by analytic hierarchy process (AHP) and Likert scale. BMC Med. Res. Methodol..

[26] Improta G., Converso G., Murino T., Gallo M., Perrone A., Romano M. (2019). Analytic Hierarchy Process (AHP) in Dynamic Configuration as a Tool for Health Technology Assessment (HTA): The Case of Biosensing Optoelectronics in Oncology. Int. J. Inf. Technol. Decis. Mak..

[27] Improta G., Russo M.A., Triassi M., Converso G., Murino T., Santillo L.C. (2018). Use of the AHP methodology in system dynamics: Modelling and simulation for health technology assessments to determine the correct prosthesis choice for hernia diseases. Math. Biosci..

[28] Improta G., Simone T., Bracale M. HTA (Health Technology Assessment): A means to reach governance goals and to guide health politics on the topic of clinical risk management. Proceedings of the World Congress on Medical Physics and Biomedical Engineering.

[29] Cesarelli M., Romano M., Bifulco P., Improta G., D’Addio G. Prognostic decision support using symbolic dynamics in CTG monitoring. Proceedings of the 13th EFMI Special Topic Conference Data and Knowledge for Medical Decision Support.

[30] Romano M., D’Addio G., Clemente F., Ponsiglione M.A., Improta G., Cesarelli M. (2014). Symbolic dynamic and frequency analysis in foetal monitoring. IEEE Comput. Soc..

[31] Romano M., Bifulco P., Ponsiglione A.M., Gargiulo G.D., Amato F., Cesarelli M. (2018). Evaluation of floatingline and foetal heart rate variability. Biomed. Signal Process. Control.

[32] Ricciardi C., Cuocolo R., Cesarelli G., Lorenzo U., Giovanni I., Domenico S., Valeria R., Elia G., Maria C.L., Mario C., Henriques J., Neves N., de Carvalho P. (2019). Distinguishing Functional from Non-functional Pituitary Macroadenomas with a Machine Learning Analysis. Mediterranean Conference on Medical and Biological Engineering and Computing.

[33] Stanzione A., Ricciardi C., Cuocolo R., Romeo V., Petrone J., Sarnataro M., Mainenti P.P., Improta G., De Rosa F., Insabato L. (2020). MRI Radiomics for the Prediction of Fuhrman Grade in Clear Cell Renal Cell Carcinoma: A Machine Learning Exploratory Study. J. Digit. Imaging.

[34] Ricciardi C., Cantoni V., Improta G., Iuppariello L., Latessa I., Cesarelli M., Triassi M., Cuocolo A. (2020). Application of data mining in a cohort of Italian subjects undergoing myocardial perfusion imaging at an academic medical center. Comput. Methods Programs Biomed..

[35] Romeo V., Cuocolo R., Ricciardi C., Ugga L., Cocozza S., Verde F., Stanzione A., Napolitano V., Russo D., Improta G. (2020). Prediction of tumor grade and nodal status in oropharyngeal and oral cavity squamous-cell carcinoma using a radiomic approach. Anticancer Res..

[36] Ricciardi C., Amboni M., De Santis C., Ricciardelli G., Improta G., D’Addio G., Cuoco S., Picillo M., Barone P., Cesarelli M. Machine learning can detect the presence of Mild cognitive impairment in patients affected by Parkinson’s Disease. Proceedings of the IEEE International Symposium on Medical Measurements and Applications (MeMeA).

[37] Sunder M.V., Kunnath N.R. (2020). Six Sigma to reduce claims processing errors in a healthcare payer firm. Prod. Plan. Control.

[38] Arafeh M., Barghash M.A., Haddad N., Musharbash N., Nashawati D., Al-Bashir A., Assaf F. (2018). Using Six Sigma DMAIC Methodology and Discrete Event Simulation to Reduce Patient Discharge Time in King Hussein Cancer Center. J. Healthc. Eng..

[39] Improta G., Balato G., Ricciardi C., Russo M.A., Santalucia I., Triassi M., Cesarelli M. (2019). Lean Six Sigma in healthcare: Fast track surgery for patients undergoing prosthetic hip replacement surgery. TQM J..

[40] Ricciardi C., Balato G., Romano M., Santalucia I., Cesarelli M., Improta G. (2020). Fast track surgery for knee replacement surgery: A lean six sigma approach. TQM J..

[41] Improta G., Guizzi G., Ricciardi C., Giordano V., Ponsiglione A.M., Converso G., Triassi M. (2020). Agile Six Sigma in Healthcare: Case Study at Santobono Pediatric Hospital. Int. J. Environ. Res. Public Health.

[42] Ricciardi C., Sorrentino A., Improta G., Abbate V., Latessa I., Perrone A., Triassi M., Orabona G.D. (2020). A health technology assessment between two pharmacological therapies through Six Sigma: The case study of bone cancer. TQM J..

[43] Ponsiglione A.M., Ricciardi C., Improta G., Orabona G.D.A., Sorrentino A., Amato F., Romano M. (2021). A Six Sigma DMAIC methodology as a support tool for Health Technology Assessment of two antibiotics. Math. Biosci. Eng..

[44] Latessa I., Ricciardi C., Jacob D., Jónsson H., Gambacorta M., Improta G., Gargiulo P. (2021). Health technology assessment through Six Sigma Methodology to assess cemented and uncemented protheses in total hip arthroplasty. Eur. J. Transl. Myol..

[45] Ricciardi C., Gubitosi A., Lanzano G., Parisi S., Grella E., Ruggiero R., Izzo S., Docimo L., Ferraro G., Improta G. (2021). Health technology assessment through the six sigma approach in abdominoplasty: Scalpel vs electrosurgery. Med. Eng. Phys..

[46] Ricciardi C., Gubitosi A., Lanzano G., Pieretti G., Improta G., Crisci E., Ferraro G.A., Jarm T., Cvetkoska A., Mahnič-Kalamiza S., Miklavcic D. (2020). The Use of Six Sigma to Assess Two Prostheses for Immediate Breast Reconstruction. 8th European Medical and Biological Engineering Conference, Proceedings of the EMBEC 2020, Portorož, Slovenia, 29 November–3 December 2020.

[47] Ponsiglione A.M., Ricciardi C., Scala A., Fiorillo A., Sorrentino A., Triassi M., Dell’Aversana Orabona G., Improta G. (2021). Application of DMAIC Cycle and Modeling as Tools for Health Technology Assessment in a University Hospital. J. Healthc. Eng..

[48] Vander Poorten V., Uyttebroek S., Robbins K.T., Rodrigo J.P., de Bree R., Laenen A., Saba N.F., Suarez C., Mäkitie A., Rinaldo A. (2020). Perioperative Antibiotics in Clean-Contaminated Head and Neck Surgery: A Systematic Review and Meta-Analysis. Adv. Ther..

[49] Improta G., Ricciardi C., Borrelli A., D’alessandro A., Verdoliva C., Cesarelli M. (2019). The application of six sigma to reduce the pre-operative length of hospital stay at the hospital Antonio Cardarelli. Int. J. Lean Six Sigma.

[50] Hackett N.J., De Oliveira G.S., Jain U.K., Kim J.Y. (2015). ASA class is a reliable independent predictor of medical complications and mortality following surgery. Int. J. Surg..

[51] Wolters U., Wolf T., Stutzer H., Schroder T. (1996). ASA classification and perioperative variables as predictors of postoperative outcome. Br. J. Anaesth..

[52] Liu S.A., Tung K.C., Shiao J.Y., Chiu Y.T. (2008). Preliminary report of associated factors in wound infection after major head and neck neoplasm operations--does the duration of prophylactic antibiotic matter?. J. Laryngol. Otol..

[53] Lotfi C.J., Cavalcanti Rde C., Silva A.M., Latorre M.D., Ribeiro K.D., Carvalho A.L., Kowalski L.P. (2008). Risk factors for surgical-site infections in head and neck cancer surgery. Otolaryngol. Head Neck Surg..

[54] Ma C.Y., Ji T., Ow A., Zhang C.P., Sun J., Zhou X.H., Wang L.Z., Sun K.D., Han W. (2012). Surgical site infection in elderly oral cancer patients: Is the evaluation of comorbid conditions helpful in the identification of high-risk ones?. J. Oral Maxillofac. Surg..

[55] Becker G.D., Parell J., Busch D.F., Finegold S.M., Acquarelli M.J. (1978). Anaerobic and Aerobic Bacteriology in Head and Neck Cancer Surgery. Arch. Otolaryngol..

